# Punishing Mothers for Men’s Violence: Failure to Protect Legislation and the Criminalisation of Abused Women

**DOI:** 10.1007/s10691-021-09455-5

**Published:** 2021-05-05

**Authors:** Sarah Singh

**Affiliations:** Lecturer in Law, School of Law and Social Justice, University of Liverpool, Chatham Street, L69 7ZR Liverpool, UK

**Keywords:** Child death, Crime, Domestic abuse, Failure to protect, Motherhood

## Abstract

This article explores the gender dynamics of ‘causing or allowing a child to die’, contrary to the Domestic Violence, Crime and Victims Act 2004, section 5. This offence was intended to allow for prosecution where a child had been killed and it was uncertain who had killed him/her, but also to allow for prosecution of non-violent defendants who failed to protect him/her. More women than men have been charged and convicted of this offence signifying a reversal of usual patterns of prosecution and conviction. This analysis interrogates how section 5 criminalises women who have experienced domestic abuse. Drawing on a case observation, reported cases and media reports of cases, I suggest this offence derives from and perpetuates patriarchal constructs of motherhood. Grounded in a feminist approach building on women’s concrete experiences of law, I conclude that section 5 should be amended so that it is only used where it cannot be ascertained which defendant actively harmed a child.

## Introduction

This article challenges the criminalisation of abused women who ‘fail to protect’ their children. It is specifically concerned with cases where fathers or male partners kill children and the mother is then charged with ‘causing or allowing a child or vulnerable adult to die or suffer serious physical harm’ contrary to section 5 of the Domestic Violence, Crime and Victims Act (as amended by the Domestic Violence, Crime and Victims (Amendment) Act 2012, s 1).

Crown Prosecution Service (CPS) data demonstrates that since section 5 came into effect in England and Wales in October 2005, 153 women have been charged with this crime, compared with 116 men (CPS 2018).^1^ This is worthy of attention, since perpetrators of violent offences are overwhelmingly male (Naffine 1997, 6; Stubbs 2016, 38; Kyd et al. 2017, 969; Elkin 2019). While the available data is not sufficiently nuanced to give an exact sense of the impact this offence has on abused women, previous studies have found that in up to 79% of cases of child death there is also domestic abuse against the mother, meaning that the majority of women charged are likely to have suffered intimate partner violence and abuse (Rabin 1995, 1109).^2^

This article explores why more women are charged with and convicted of this offence, providing the first in-depth feminist critique of sections 5 and 6 of the Domestic Violence, Crime and Victims Act 2004. In so doing, it considers the ways in which abused women are criminalised under these provisions, both in terms of how the offence was drafted and how it is interpreted in the courtroom. Particular attention is paid to how these provisions criminalise mothers who are themselves survivors of domestic abuse.

First, I outline the offence and explain its genesis. Second, engaging with Hansard debates concerning the introduction of this offence, I argue that section 5 derives from gendered constructs of risk and responsibility. Criminalising abused women for failing to protect their children is one of the more blatant examples of what Stanko terms the “explicit message of the ‘criminology of the self’ warning us (women) to be good citizens by avoiding men’s violence” (Stanko 1997, 490). The responsible mother is diametrically oppositional to constructs of the vulnerable victim, allowing legislators to presume (and later prosecutors to allege) that women stay in abusive relationships out of fecklessness, betraying dangerous ignorance around the dynamics of abusive relationships.

‘Failure to protect’ cases are primarily heard in the Crown Court, meaning that they are typically unreported.^3^ Due to the paucity of reported cases, my doctrinal research on primary sources was supplemented by stories reported in the media and enriched by observation of one case in its entirety. Having engaged with the limited reported case law, I then undertook an analysis of section 5 cases reported in national, regional and local newspapers. Local newspapers proved a particularly rich source, as they frequently publish what are effectively running commentaries on cases of local interest. These often include direct quotations of judicial commentary and, in high profile cases such as homicides, Judge’s sentencing remarks in full (as encouraged by the government in attempts to effectively communicate the outcomes of criminal trials to local communities (Ministry of Justice 2011)). The relatively recent ability of journalists to live-blog proceedings directly from the courtroom, coupled with the relaxation of word counts/pages due to online publishing, means that it has been possible to analyse extracts of transcribed narratives. Given the limitations of official law reporting, the value of media reports in revealing gendered court room discourses is well established in socio-legal studies. For example, Fox and Bell (1996), Weare (2013) and Cunliffe (2011) all draw upon media reports of cases to explore tropes pertaining to women in the criminal justice system. Although it is prudent to exercise caution when relying on media reports of cases, my findings revealed that media accounts, while often more focused on women who failed to prevent harm than men who caused it, were not particularly sensationalist (Goc 2007; Cunliffe 2011). This is possibly due to the dynamic in many cases being somewhat banal, in that they generally featured a male aggressor who resented caring responsibilities and a female partner constructed as passive and abused.

In the course of the media search, I became aware of the proceedings in *R v Green & Critchley.*^4^ Grounded in a feminist approach building on women’s concrete experiences of law, I decided to observe this trial at Preston Crown Court in its entirety since it appeared to be a classic example of how this offence operates in the context of abused women charged with this offence.^5^ As part of daily reflections on my observations, I would check that day’s media reporting against my observational notes. This process confirmed that the press coverage was a faithful representation of what had happened that day in court*. Green & Critchley* is emblematic in that it exemplifies all the key themes and accords with reported cases in both law reports and the media. Furthermore, in-depth case observation provided additional insight into how section 5 is mobilised in practice and how procedural rules impact women charged with this offence.

Drawing upon the above sources, I demonstrate that, in assessing whether women knew or ought to have known of the risk posed by their partner, a history of domestic abuse is used against them, even when no evidence exists that the partner was violent towards the child. This silences women, rendering them unable to disclose abuse they have suffered for fear of being convicted. I then argue that when assessing whether women failed to take reasonable steps to mitigate against that risk, gendered expectations of care mean that the reasonable steps expected of mothers are set much higher than for fathers accused of failing to protect their children. Masochistic expectations of mothers, coupled with ignorance regarding barriers to leaving abusive relationships, means that a failure to leave is conflated with a failure to protect. Finally, arguments for creating a ‘battered woman’ defence are assessed, but ultimately dismissed in favour of amending this offence so that it only applies in circumstances where it is genuinely unascertainable who, from a pool of possible perpetrators, inflicted the harm.

## Entwining criminal and maternal responsibility

Feminist criminological theory shows us that crime is an inherently gendered concept. Women who commit crime are ‘doubly transgressive’ in challenging both the criminal law and their gender role (Carlen and Worrall 1987; Worrall 1990). Thus, when women commit crime they are tried as women and mothers, not as autonomous legal subjects (Naffine 1997, 144). Consequently, mothers accused of crimes are effectively convicted or acquitted according to their adherence to maternal ideology, rather than their actions and omissions.

In interrogating the gendered nature of this offence, I argue that section 5 derives from patriarchal constructs of the ideal mother. Influential eighteenth century philosophers, such as Jean Jacques Rousseau, were instrumental in positioning mothers as responsible for the welfare of children and, ultimately, society (Badinter 1980). More contemporaneously, the impact of neoliberal privatisation of care has led to expectations of intensive mothering, which re-emphasise the demand for maternal omnipresence and selflessness (Hayes 2005). The naturalisation, then later normalisation, of these conventional constructs of mothers have ultimately led to a contemporary culture of mother blaming which means that when tragedy befalls children, mothers are presumed blameworthy (Smart 1996; Ward 2004, 369).

The expectation of maternal omnipresence plays a key role in prosecution contentions that the mother ought to have foreseen a risk of harm (and been there to prevent it). Moreover, expectations of maternal selflessness mean that engagements in paid work outside of the home are employed effectively by prosecutors to convince juries that women are both morally and legally culpable. As will be seen, conventional expectations of mothers as enshrined in ‘failure to protect’ provisions allow both moral and legal focus to be shifted on to the woman who failed to prevent the harm rather than the man who caused it. In scrutinising mothers who fail to prevent harm, rather than the abuser who inflicted it, this ideology has a disciplinary effect on women which, due to media reporting of crime, reaches far beyond the confines of the courtroom.

Moreover, this ideal responsible mother of social discourse has influenced and become entwined with the responsible subject of criminal law. The nebulous concept of responsibility remains at the heart of both what makes the ideal mother and the criminal legal subject. However, this language of responsibility, risk and blame takes on a new significance under neoliberalism, particularly in an ongoing age of austerity (de Benedictis 2012). Patriarchal values continue to be embedded in neoliberalism and the neoliberal language of ‘risk’ continues to dominate socio-legal responses to both crime and motherhood. This reiterates and exacerbates the feminisation of care (Cain 2016b) and crime prevention (Stanko 1997, 480). In contemporary western society, it is not only women’s responsibility to care for children, but also to avoid and manage male violence. Due to the masculinisation of risk, risk avoidance is part of femininity and also motherhood (Hannah-Moffat 2009). Good women and mothers do not take risks (Walklate 1997). Risk management is simply another aspect of gender performativity required of the ideal woman and mother (Gardner 1995). Conventional constructs of motherhood and risk have led to a ‘deficit’ model of motherhood (Lapierre 2008, 454), whereby if anything undesirable happens to a child, her mother is culpable. This culture is so pervasive that, as shown in the cases analysed in this article, women are blamed for the actions of men; the cultural and legal focus is on their failure to stop the violence, rather than on the infliction of harm.

## The history and motivations behind criminalising ‘causing or allowing a child (or vulnerable adult) to die or suffer serious physical harm’

Section 5 is truly a product of neoliberalism; it is one of the New Labour Government’s (numerous) new criminal offences introduced as part of their ‘tough on crime, tough on the causes of crime’ agenda (Reiner 2007). In its entirety, the Domestic Violence, Crime and Victims Act 2004 was lauded by the then Home Secretary as “the biggest overhaul of domestic violence legislation in several decades” (United Kingdom Hansard 2004a, col. 536). It emphasised ‘putting victims first’ and challenging ‘unacceptable past social norms’ which, as Parliament recognised, were often barriers to seeking help. It is paradoxical, therefore, that the statute contains provisions which criminalise the actions of women who are themselves victims of domestic abuse. Section 5 was initially introduced to remedy a lacuna resulting from *R v Lane and Lane* (1986),^6^ in which it was held that where a child had been killed and it was impossible to discern who out of a potential pool of defendants had actively harmed the child, the case could not proceed. Failed prosecutions in a number of child deaths sparked outrage both amongst the public and child protection groups, notably the National Society for the Prevention of Cruelty to Children (NSPCC), and prompted a Law Commission consultation in April 2003 (Law Commission 2003).

However, the offence of ‘causing or allowing a child to die or suffer serious physical harm’ goes much further than allowing a means of prosecution when the perpetrator is unidentifiable. Section 5 was drafted as a negligence-based offence designed to apply in situations where it is clear from the outset which party is the active abuser, and which party failed to protect the child by ‘allowing’ the abuse to happen. Consequently, so-called passive abusers can be charged with a homicide offence, even when it has been established that they did not actively harm the child. Initially, the offence was ‘causing or allowing the death of a child or vulnerable adult’. It was amended in 2012 to encompass situations where a child or vulnerable adult has incurred serious injury^7^:

5(1) A person (“D”) is guilty of an offence if:

- a child or vulnerable adult (“V”) dies [or suffers serious physical harm]^8^ as a result of the unlawful act of a person who:
  (i) was a member of the same household as V, and
  (ii) had frequent contact with him,
- D was such a person at the time of that act,
- at that time there was a significant risk of serious physical harm being caused to V by the unlawful act of such a person, and
- either D was the person whose act caused *V's death* [the death or serious physical harm] or:
  (i) D was, or ought to have been, aware of the risk mentioned in paragraph (c),
  (ii) D failed to take such steps as he could reasonably have been expected to take to protect V from the risk, and
  (iii) the act occurred in circumstances of the kind that D foresaw or ought to have foreseen.

## Using the threat of criminalisation to ‘help’ women leave abusive relationships

Section 5 imposes a duty to protect on any member of the household who is over 16 and has frequent contact with the child (unless they are the child’s parents, in which case they can be charged with this offence even if they themselves are a child under 16).^9^ The debates around this definition alone provide valuable insight into who Parliament considers responsible for children (and vulnerable adults) and, equally importantly, who evades liability. I highlight how, when drafting this offence, Parliament imposed a legal duty on women who they acknowledged were likely to themselves be subject to interpersonal violence and abuse. In this article, I limit my discussion to how this offence particularly disadvantages mothers.

Initially both Houses of Parliament expressed reservations about imposing an obligation to protect on abused women. However, this duty was ultimately justified on the misguided basis that criminalising a failure to protect would support women to leave abusive relationships:

We need a legal culture that sustains and supports the victim far better than the one we have at the moment. There is a feeling among many victims that they do not have the capacity to get themselves out of the situation in which they find themselves. We must find ways, either through the agencies with which we engage or the legislation that we introduce, to reach out to those people and tell them that there is a way in which they can get themselves out of their relationships and that the law and society will support and help them in the future (UK Hansard 2004a, col. 555).

It is noteworthy that while this sentiment dominates the narratives of several parliamentarians (United Kingdom Hansard 2004a, col. 536–615), not one proceeded to explain how the threat of criminalisation assists women with the risky process of leaving an abusive relationship. This highlights their lack of understanding of the additional barriers that having children places on leaving an abusive relationship. This is particularly pertinent given that 30% of intimate partner violence is estimated to begin during pregnancy (Mezey and Bewley 1997; United Kingdom Hansard 2004a, col. 537).

In the consultation process preceding enactment of this offence, Refuge argued that it was crucial for Parliament to appreciate ‘the real risk involved in leaving – a person’s reasonable fear she will be killed’ (United Kingdom Hansard 2004b, col. 353). Leaving a violent relationship is equally hazardous for children. If a woman does leave the relationship, she then faces a potential custody battle with her abusive ex-partner. Women’s allegations of domestic abuse in custody battles continue to be viewed with suspicion (Barnett 2015). In the family court, she risks allegations of violence being dismissed as the claims of an ‘implacably hostile mother’, a trope which depicts women who oppose shared custody as seeking to alienate their child’s father for their own selfish complex motives. This is a contention all too frequently levelled at women who claim they suffered domestic abuse and may result in the abuser being granted unsupervised contact (Wallbank 1998, 357). In such circumstances “leaving becomes ‘failure to protect’ rather than a form of child protection” (Stark 1995, 973).

When men kill their children, it is often in circumstances when their relationships are threatened or have broken down (Leveillee et al. 2007). Women’s Aid recently highlighted the plight of nineteen children murdered by their fathers over the last decade (Women’s Aid 2016). All of these men were granted unsupervised contact, despite warnings from the child’s mother. This has led to calls for reform of how the family courts respond to the plight of abused women and children (Choudhry 2019). The logic of ‘failure to protect’ discourse in the courtroom thus fails to appreciate the numerous risks associated with leaving an abusive relationship. This not only includes endangering women and their children but also other risks including destitution, homelessness and loss of support networks.

As a result, it is impossible to criticise this provision without acknowledging government policies which impact the lives of those most likely to be criminalised under this offence. While an in-depth exploration of socio-economic context is beyond the confines of this article, it is worth noting that shortly after the introduction of this offence the UK entered an ongoing period of austerity. Cuts to public spending have disproportionately impacted women, and particularly single mothers (Karamessini and Rubery 2014; Cain 2016a). Despite a rise in domestic homicide, fewer refuges exist than when the offence was introduced and 75% of local authorities in England slashed their spending on domestic violence refuges by nearly a quarter (24%) between 2010 and 2017 (Women’s Budget Group 2019a). Moreover, the lack of social housing, the two-child benefit policy and the numerous problems with universal credit (particularly the payment to one partner only) all directly impact on women’s ability to leave abusive relationships (Cain 2016a, 488; Women’s Budget Group 2019b). Put simply, government policy has created a climate where it is probable that increasing numbers of women will be convicted of this offence. This claim is substantiated by the above statistics which have generally shown a year on year increase in the women charged and convicted under section 5 (CPS 2018).^10^ Thus the purported ability of this provision to enable women to leave abusive relationships is simultaneously eroded by other legal, social and political changes.

The imposition of this duty to protect on women who are abused by a new partner who is not the child’s father is particularly problematic given Parliament’s decision to exonerate disengaged or absent fathers. These fathers appear to evade culpability merely on the grounds that they may have a difficult relationship with the mother:

…Absent fathers may also have a duty of care but I do not believe that they should be included in this offence. Such people are in an arguably more difficult position. The father may have a difficult relationship with the mother. To place on that father a duty to take reasonable steps to safeguard his child may only increase the pressure on an already difficult situation. (United Kingdom Hansard 2004b, col. 361).

The belief that “it is a parent’s fundamental responsibility to protect children” (United Kingdom Hansard 2004c, col. 74) also means that children aged under 16 can be charged with this offence if they are the victim’s parents. However, as the defendant must also be a member of the victim’s household this again has gendered implications since, where parents have separated, children are more likely to reside with their mothers, with 90% of single parent families being headed by mothers (ONS 2019).

## Coercive control of abused mothers: Forcing women to testify against their abusers

Alongside the introduction of a new homicide offence, section 6 of this legislation also enacts procedural changes proposed to remedy the legacy of *R v Lane and Lane*; namely the drawing of negative inferences from silence and postponing a finding of no case to answer (Clayton-Helm 2014, 477).

Charging abused women with a homicide offence is intended to encourage them to testify against their partners, thus helping the prosecution to secure a murder conviction against their co-defendant. The coercive effect of these procedural changes was articulated by Dominic Grieve in the debates around the offence’s introduction:

The Government are seeking to establish what I can only describe as a double whammy of procedure and evidence …in the hope that defendants will be coerced or feel under an obligation to give evidence in the witness box because they face the double whammy. Although they might not wish to give evidence because they know that then they would be unlikely to be convicted of murder, but they may wish to give evidence because without it they are certain to be convicted of the negligence offence [s 5]. This is the double whammy; if one does not go down one road, one renders oneself likely to suffer a worse outcome on the other. That is the Government’s intention, and what their proposal is designed to achieve.^11^

The intentionally coercive ‘pincer movement’ orchestrated by sections 5 and 6 together is also acknowledged by the Home Office in their guidance regarding the Act:

The prospect of an adverse inference being drawn in relation to murder or manslaughter as well as the new offence [s.5], causing or allowing the death or serious harm to a child/ vulnerable adult] may encourage one or more parties to give evidence explaining what happened (Home Office 2005a).

The Home Office guidance states that juries may only draw an inference if the judge directs that it is “proper to do so; the[y] must acquit if they think the defendant’s silence is attributable to another reason not guilt” (Clayton-Helm 2014, 477). This is intended to safeguard against the drawing of negative inferences where silence is prompted by reasons other than hiding information; for example, where a defendant is genuinely too afraid to testify. In practice however, judges and juries will interpret a defendant’s motive for remaining silent according to patriarchal notions of appropriate femininity (Cunliffe 2011; Edwards 2012). A failure to incriminate one’s partner is taken as indicative of guilt. It is presumed that women who refuse to incriminate their partners do so out of fecklessness, not out of fear or a genuine belief that their partner would not have done that of which they are accused.

Allowing negative inferences to be drawn from a defendant’s silence conflates a failure to account with a ‘failure to protect’. Section 6 derives from and perpetuates the assumption that a defendant who fails to provide an account only does so because they are duplicitous and/or deliberately obstructive. As a result, any contradictory remarks are used to call a woman’s credibility into question. Rather than noting how context (and emotion) may result in inconsistencies between a woman’s original statement and her courtroom narrative, women are frequently treated as ‘hostile witnesses’ (Edwards 2012; Porter 2019). This ignores the well-established finding that victims of domestic abuse may be reluctant to support prosecution of their abuser due to fear of further violence, either from the abuser or their associates (Edwards 2012). In reality, “a witness who is in fear may be as effectively unavailable as a witness who is dead, ill or overseas.”^12^ Moreover, it also presumes that the ‘passive’ abuser charged under section 5 was present when the serious physical harm was inflicted. This, however, was not the case in the vast majority of cases analysed in this article. The plight of vulnerable defendants is further compounded by reductions in legal aid spending, driven by a neoliberal ideology of public sector austerity. This has resulted in increasingly poor-quality legal representation in police stations due to a drop in numbers of duty solicitors following funding cuts (Smith and Cape 2017).

By eroding a defendant’s right to silence, this provision allows ideological constructs of the ‘good’ mother to play a prominent role in the disposition of these cases. Allowing juries to presume guilt from a failure to incriminate one’s partner permits mother blaming to flourish. It also exemplifies how sections 5 and 6 derive from and perpetuate the victim-blaming culture which this statute was, in part, intended to overcome.

## Foreseeability and risk as gendered constructs

When looking specifically at the plight of abused mothers, a particularly troubling aspect of this offence is the requirement that the defendant accused of allowing harm to come to the child must ‘have known or ought to have known’ of the ‘risk of serious physical harm’. The judgment in *R v Khan*^13^ makes it clear that “[i]t is the foresight of the kind of harm which is fundamental to the defendant’s liability rather than the awareness of method/type of harm effected by the individual who carries out the actus reus of the crime” (Reed 2010, 200). Consequently, a defendant’s foresight of risk is assessed according to a qualified objective test. Essentially the jury is asked to assess the defendant’s actual calculation of risk, then to decide if that aligns with a reasonable person’s calculation of risk in that context. Enshrining this subjective element led some academics who had welcomed the enactment of this offence to downplay concerns regarding the criminalisation of abused women (Morrison 2013, 826). Morrison, for example, argued that if a woman’s assessment of risk was influenced by having experienced domestic abuse she would not be convicted, as she would not be found to have been aware of the risk (Morrison 2013).

However, as criminologists such as Walkate and Stanko recognise, risk is a gendered construct frequently mobilised to responsibilise women (Walklate 1997; Stanko 1997). This offence is an obvious example of this process: while foreseeability of harm appears gender neutral, in practice, gendered expectations of care render this anything but. This assessment of risk is also based on the idealised middle-class mother, taking no account of factors such as age, class, race, socio-economic status etc. (Moffat 2018). It is argued that this offence is an example of the state punishing women for ‘getting it wrong’ (Stanko 1997). Mothers who are charged under section 5 have simultaneously failed as women, in that they have failed to avoid or manage the risk of male violence, and failed as mothers, as they have not been omnipresent thereby ensuring their child’s welfare at all times.

Evidence suggests that the general public find women more culpable for failing to protect their children if they have experienced violence (Worden and Carlson 2005, 1219). This culture of mother-blaming is so pervasive that victims of domestic violence are no less likely to blame a mother for allowing her child to come to harm than women who have never experienced intimate partner violence (Weisz and Wiersma 2011, 419). Consequently, to play to the jury when mothers are on trial for failing to protect their children, prosecutors may argue that the more frequent or severe the abuse suffered by the mother the more she ought to have known of her partner’s propensity towards violence and have mitigated against the risk posed by them (Chan and Rigaros 2002, 105; Herring 2007, 2008). Thus, rather than domestic violence mitigating culpability, in practice, jury's interpretations of the objective element cause women to be convicted *because* of the abuse they have suffered. For example, on 8th March 2012, Kirsty Smedley, 24, alleged that her partner Daniel Rigby, 23, had kicked her in the face whilst she was pregnant. On 13th of March, she stated that he had dragged her, pushed her head into a door and head-butted her in public. After this assault, it was reported that she left the family home with their two-year-old son, Rio. However, she quickly returned as Rigby followed her, threatening that if she refused to come home he would “batter [her] proper” (Dobson 2014). Upon returning home he assaulted her again, punching her in the side of the head while demanding money. Smedley was persuaded by her midwife to report Rigby to the police when she confided that she thought Rigby “would kill her” (Dobson 2014). Two weeks later Rigby killed Rio. While it was accepted that Smedley was absent from the family home when Rigby fatally attacked Rio, the prosecution argued that Smedley ought to have foreseen the possibility of violence against Rio and failed to prevent it out of fecklessness rather than fear: “What she was fearful of wasn’t what might happen to her child but what might happen to her if she didn’t please him. Rio was very much second in her thoughts. He was not her priority (Dixon 2012)”.

Rather than intimate partner violence counting as mitigation, it served to condemn Smedley. The prosecutorial narrative convinced the jury, resulting in her conviction for causing or allowing her son’s death, for which she received a 4-year prison sentence.^14^

Courtroom observation of *R v Green and Critchley* similarly highlighted how prosecutors may argue that the more frequent or severe the abuse suffered by the mother, the more she ought to have known of her partner’s propensity towards violence. In this case, prosecutors sought to rely upon an incident *after* their daughter Lia’s death in which Green was seen kicking and punching Critchley on a bus in order to prove that she ought to have foreseen the risk of harm to Lia:

There is also evidence that Richard Green was capable of assaulting Natalie. There was an incident on a bus, six weeks after Lia’s death where Green had lost his temper and physically assaulted Natalie in an uncontrolled and uninhibited way in front of witnesses. *This may have occurred post-death, but it may be a fair inference that he has physically abused her in the past. Furthermore, Natalie told the bus driver; ‘he is always like that’. Thus, it is suggested, Natalie would have been aware of Richard Green’s propensity to violence and the jury could be invited to consider that she should have taken steps to protect Lia - either by removing her from his care and taking her to a place of safety or by managing things within the household so that he was not alone with Lia and able to commit his wicked assault.*^15^

This prosecutorial tactic of using domestic abuse to prove a defendant’s culpability is paradoxical given the aims of the Domestic Violence, Crime and Victims Act 2004 as a whole. This approach also appears irreconcilable with CPS guidance around this offence which specifically advises that prosecutors be mindful of myths and stereotypes around domestic abuse that derive from and perpetuate a culture of victim-blaming (CPS 2019a).

Moreover, while I do not wish to pathologise victims of abuse, the effect of sustained abuse is implicitly coercive. This potentially affects the victim’s agency, impacting her calculation of risk and perception of what is reasonable in the circumstances (Mahoney 1991; Carlton and Krane 2013). While women such as Critchley and Smedley are expected to calculate that violence towards them will lead to violence towards their children, the same logic does not extend to calculations of risk undertaken in the family courts. In *LBC v Gray and Butler* (2012)^16^ when returning Ellie Butler into the care of her violent father and abused mother, Lady Justice Hogg failed to understand Ben Butler’s prior violence towards an adult as inferring risk to a child: "[Ben Butler] acknowledged his criminal convictions and said there were some things he regretted and was not proud of… I note the convictions are on adults, not on children" (627).

As acknowledged in *R v Lee* (Unreported 2016), to accurately assess a defendant’s appreciation of risk, a jury needs a clear understanding of the context surrounding her actions and choices. The court acknowledged this by clarifying that in assessing what a defendant could have foreseen it is appropriate to consider factors such as her “age, life experience and experience of the co-defendant” (Freer 2016, 623). Yet, as we have seen, any history of interpersonal abuse is deployed to convict women charged with this offence, meaning that defence barristers may downplay, or even omit, abuse which their client suffered at the hands of her partner. Thus, in *R v Green and Critchley,* both defence barristers jointly petitioned the judge for further evidence of domestic violence to be omitted from open court, since it would prejudice both parties. Responding to Green’s defence plea to exclude evidence of domestic violence, Critchley’s barrister, explained; “I supported it as the jury would draw inferences from the fact that he is violent towards her…”^17^ The judge agreed. While this was to Critchley’s advantage, as she could not be condemned on account of the abuse she herself had suffered at the hands of Green, effectively this served to silence her. During the remainder of the trial she could not put her actions or omissions in context as she had to erase her experiences of violence to avoid incriminating herself.

Denying women the opportunity to disclose a history of the abuse is problematic for several reasons. First, it leaves unexplained gaps in a woman’s testimony. In turn this makes her (and any supporting witnesses) appear disingenuous. To a jury, her narrative does not make sense and they interpret her as having a dishonest motive in “hiding information” (Nadler 2012, 1). This can play a key part in women’s conviction, as juries seek to punish only risk takers who they judge to be morally culpable (Weait 2007). Construing women as duplicitous legitimises the silencing of women who have offended, particularly where they are co-defendants, as is often the case in section 5 trials (Barlow 2015, 469). Secondly, as I have written elsewhere, denying women the opportunity to disclose the context in which they were parenting creates gaps in courtroom narratives which are plugged by archaic tropes of female criminality (Singh 2017, 511). Finally, silencing mothers with the result that they cannot contextualise their omissions means that their actual appreciation of risk remains unknown. Rendering a mother’s real appreciation of risk unknowable means more weight is given to the objective standard: what she ought to have foreseen. Silencing women in this way exacerbates the woman’s vulnerability to conviction because the lack of factual context results in increased reliance on misogynistic tropes of female criminality and idealised notions of motherhood (Seal 2010; Cunliffe 2011). The reliance on these tropes allows for no subjectivity or engagement with women’s real lived negotiation of risk when parenting in abusive relationship (Stanko 482).

This supports the criminalisation of abused mothers since the high level of responsibility and omnipresence expected of mothers means that the taking of *any* risk is liable to be perceived as immoral, thereby inviting criminalisation (Friedman 2014, 226). Reliance on this objective standard also raises the concern that when mothers are on trial their behaviour is compared to the ideal mother, not the reasonable man, as I now explore.

## The reasonable steps expected of the ideal mother

To be convicted of the section 5 offence a defendant must have not only foreseen or ought to have foreseen the risk to the child, but also have failed to take ‘reasonable steps’ to mitigate against this harm. As with other aspects of this offence, it is for the jury to determine what could be reasonably expected of the defendant at that time, thus making this a qualified objective test.

It is clear from the judgment in *R v Khan* [2009]^18^ that special consideration should be given in situations where defendants are themselves victims of abuse. Moreover, Lord Judge clarified that domestic violence and its impact will be taken into account when deciding whether a defendant took reasonable steps to protect the victim.^19^ However, as established above, evidence of domestic violence is often omitted in court as it can be used to bolster prosecutorial arguments that the defendant knew of her partner’s propensity to violence. As a result, the context in which her actions—or, more accurately, her omissions—occurred is often absent from court narratives.

I argue that when mothers are accused of this offence, ‘reasonable’ becomes conflated with ‘ideal’ (Fugate 2001). Juries are encouraged by prosecutors to interpret a mother’s failure to meet this idealised standard of mothering as justifying the imposition of criminal responsibility on women who have experienced abuse (Panko 1995; Herring 2008). Archaic associations between femininity and suffering mean that the good mother is required to be self-sacrificing to the point where she will die for her child (Jacobs 1998). It follows that, even where domestic abuse is disclosed, gendered expectations of mothers mean abused mothers are not exonerated from responsibility for their child’s death. Denial of situational vulnerability (meaning that experienced by those encountering circumstances of social disadvantage (Brown et al. 2017, 502)), means that the threshold of what is reasonable is set too high in these cases (Cobbe 2008). For example, *R v Thomas* (unreported 2015)^20^ concerned the murder of 11-month-old Oliver Sargent by his father, Paul Thomas. The judge acknowledged that Paul had been physically violent towards Oliver’s mother, Ashlea (Carter 2015). Despite this, Ashlea Thomas was convicted of causing or allowing her son’s death and sentenced to two years in prison, suspended for two years. Her case illustrates that, even when abuse is disclosed, there is little evidence that either judges or juries appreciate how it may affect a mother’s calculation of how best to protect her child.

In several of the cases discussed in this article, women were convicted for failing to protect their children despite being at work when the injuries to the child were inflicted. Expectations of maternal omnipresence mean that the courtroom discourse in cases such as *R v Green and Critchley*^21^ and *R v Stephens and Mujuru* (2007)^22^ contains “undertones of the irresponsible mother which would not be applied to the father who, would of course be earning the family bread” (Herring 2007, 929). Such narratives betray how women’s engagement in paid work is considered an indulgence or privilege. In contrast, male employment is considered so important that it exonerates fathers from taking responsibility for their children. This is all the more striking when the above cases are compared with the discourse and outcome in *R v Ege (Sara)* (2014).^23^

On 12th July 2010, Sara Ege called emergency services reporting that her house was on fire and her son, Yaseen, was trapped in the burning building. Yaseen was pronounced dead upon arrival at hospital. A post-mortem, however, revealed that Yaseen had not died from injuries sustained in the fire, but rather from blunt force trauma. Injuries on his body were consistent with sustained abuse over a prolonged period, including evidence of fractures to his ribs, wrist, and clavicle, finger, bruising to the head and arm, swelling to the brain and bleeding in the stomach. When interviewed by police, Sara Ege admitted abusing Yaseen, hitting him “like a dog” with a stick or pestle. She also confessed to pouring barbeque fluid on his body, explaining that “I knew he was gone but I was trying to protect myself” (BBC News 2012). Ege was sectioned and detained in a secure psychiatric unit months before trial. Four months later Ege retracted her confession saying that she had falsely confessed due to pressure from her husband and his family. She alleged abuse throughout her marriage and that Yaseen had been fatally struck by her husband when he tried to intervene upon seeing his father assault his mother.

During their trial it was established that Sara Ege had sought medical assistance on six occasions for injuries she claimed were inflicted by her husband. Testifying at her trial, her parents, who lived in India, said they were aware that she had experienced violence (including being knocked unconscious) from the beginning of her marriage. Her mother gave evidence that, following Yousef's arrest for assaulting their daughter in 2007, his family, who also lived in India, visited their home and threatened them if anything happened to Yousef. Consequently, Ege dropped the charges against her husband. After several weeks, Ege was convicted of murdering Yaseen and perverting the course of justice. She was jailed for life, serving a minimum of 17 years for murder and an additional four years for perverting the course of justice. Her appeal was unanimously dismissed.

Despite Yaseen’s post-mortem revealing evidence of prolonged and severe abuse, Yousef Ege was acquitted of causing or allowing his son’s death. Peter Birkitt QC convinced the jury that his client had no idea that his wife was abusing his son as he “had never complained to him” (Keen 2013). Furthermore, he argued successfully that, “by working as a taxi driver and then doing work around the house, Yousef did not have the advantage of schoolteachers who were with Yaseen six hours a day” (Keen 2013). Gendered understandings of employment excused Yousef Ege from caring responsibilities for his son, whereas women such as Critchley and Mujuru were held responsible for their children’s care in addition to their work outside of the home.

## Conflating a failure to leave with a failure to protect

The Home office and Ministry of Justice have issued guidance on the range of actions which may constitute ‘reasonable steps’ that a responsible adult might take to protect a child (Home Office 2005b, Ministry of Justice 2012). However, it is clear from case law that in situations of intimate partner abuse, reasonable steps are equated with leaving the relationship. This is such a dominant motif of ‘failure to protect’ discourse that a failure to leave an abusive relationship is seen as synonymous with a ‘failure to protect’.

When cases are played out in court, prosecutorial and judicial narratives betray the same ignorance of the barriers to leaving abusive relationships as shown by Parliamentarians when drafting this offence. The assumption of choice prevalent in judicial comments reveals an inability to appreciate the realities of domestic abuse, evident in early provocation cases regarding abused women such as Sara Thornton and Kiranjit Ahluwalia (Nicolson 1995).

Ignorance of the realities of mothering in a context of domestic abuse is particularly apparent from judicial narratives in *R v Green and Critchley.*^24^ Prosecutorial emphasis on the fact that Critchley’s mother lived just streets away led the judge to remark how ‘easily’ she could have left Green. Due to class-based assumptions about space and resources, he failed to consider that several adults and children already lived in Critchley’s mother’s house. It would not have easily accommodated another adult and two children. Moreover, the fact that both houses were in such close proximity makes this *more* dangerous: she could never have fled to her mother’s house without Green knowing where she and their children were residing. These cases underline judges’ consistent failure to recognise that pregnancy and the post-natal period are especially difficult times to leave an abusive relationship, emotionally and financially.

This dogged focus on leaving an abusive relationship also fails to recognise the numerous ways women endeavour to mitigate the risk presented by their partners. Critchley was employed full time as a nursery nurse, while Green was unemployed. Despite this, Critchley managed any potential risk posed by Green by, for the most part, ensuring that their children were never left in his care. The week that Lia died was not a typical week. Lia and her siblings were not permitted to attend nursery for a fortnight as Critchley had fallen into arrears with nursery fees. Most days the children had been looked after by Critchley’s mother, however on the day of her death Lia had been left with her father as both Lia and Critchley’s infant brother had stomach upsets. Similarly, in *R v Lewis* (Unreported 2016),^25^*R v Kenny (Hayley)*^26^ (Unreported 2007) and *R v Mujuru* (2007)^27^ the children were left in the care of the male partner only because the women in question needed to work and had no other option. A continuing preoccupation in court with why the woman did not leave maintains the focus on her as the party who failed to manage the risk of abuse rather than scrutinising the abuser (Magen 1999,127).

## Why a ‘battered woman’s’ defence is not a solution

Concerns regarding how this offence specifically criminalises mothers who are subject to domestic abuse has led many commentators, including Herring (2007, 2008), Midson (2014), Miccio (1999) and Hayes (2005), to argue that there should be a specific defence to ‘failure to protect’ provisions for those who have suffered intimate partner abuse. This view was endorsed by members of the House of Lords, who proposed an amendment to the initial Bill so that the offence would specifically not apply to those who were themselves victims of the abuse, unless they actively participated in inflicting the harm to the child (United Kingdom Hansard 2004d, col. 1160). The amendment was, however, rejected on the grounds that allowing those suffering intimate partner violence a defence would undermine the effectiveness of the provision. Attempts at amending the offence to recognise the correlation between child abuse and domestic abuse were rejected due to worries that taking domestic abuse into account would undermine the aims of the offence by rendering it ‘unprosecutable’ (United Kingdom Hansard 2004c, col. 74). Introducing a ‘battered woman’s defence’ was also deemed undesirable due to concerns about the privileging of domestic abuse over other mitigating circumstances. MPs, including Paul Goggins, argued that this would be inappropriate as it would “single out defendants who are vulnerable because of domestic violence from among other vulnerable defendants…those who are very young, disabled or frightened for some other reason” (United Kingdom Hansard 2004c, col. 75).

While I have argued that domestic violence should mitigate a parent’s supposed failure to protect, it does not, in my view, follow that a specific defence is needed. First, domestic abuse takes a range of forms: psychological, emotional, physical, financial and/or coercive control. Thus, introducing a specific defence based on the ‘reasonable battered mother’ risks creating a statutory or judicially-created hierarchy of domestic abuse, whereby physical violence would almost certainly be prioritised above coercive control, not least because it is easier to prove (Bishop and Bettinson 2017).^28^ The definition of domestic violence in the Domestic Violence, Crime and Victims Act 2004 is deliberately broad to encompass a wide range of abuse; however, in the courtroom, juries will revert to stereotypes of abusers and victims (Magen 1999, 130). This is problematic as coercive control is more likely to lead to a situation where a mother is unable to protect her child from harm than where there is physical abuse (Stark 1995; Lapierre 2008).

Secondly, the creation of a specific defence for victims of domestic abuse risks denying the victim’s agency (Morrison 2013). As we have seen, the equation between ‘protecting’ and ‘leaving’ already ignores the numerous ways that mothers attempt to protect their children. The case law consistently shows women selecting the least dangerous option, a decision-making process which Stark terms exercising “control in the context of no control” (Stark 1995, 973). For this reason, I also disagree with Skinazi’s argument that the defence of duress should be expanded to apply to abused women who fail to protect due to coercion (Skinazi 1997). Due to the dominance of gendered tropes in courtroom discourse, female defendants are effectively convicted or acquitted due to their adherence to gender roles rather than their actions or omissions (Seal 2010). Defences such as duress risk playing into this denial of agency as this would be likely to operate on the same gendered notions of objectivity as diminished responsibility and provocation (Quick and Wells 2006, 523).

Rather than framing a specific defence, I advocate amending section 5 so that it only applies in circumstances where it is genuinely impossible to ascertain who from a pool of perpetrators actively harmed the child. The lacuna highlighted by *R v Lane and Lane* (1986)^29^ needed to be closed and indeed section 5 has addressed this. However, allowing for prosecution of abused women to coerce them into testifying against their partner’s goes too far. At present, where a child has clearly been killed by their father or mother’s partner the default position appears to be to charge the mother with this offence and let the court decide her fate. It is envisioned that if women comply by giving an account which supports the conviction of her co-defendant, the charges against her will be dropped or she will be acquitted. Therefore, the combined effect of sections 5 and 6 is that abused women are treated as collateral damage; they are the inadvertent casualties of the criminal justice system. While parliamentary debates indicate that parliament intended this offence to be used coercively, this is inconsistent with the broader aims of the Domestic Violence, Crime and Victims Act 2004. It is therefore suggested that Parliament reconsider this offence and make it clear that prosecution of a woman who has suffered abuse is not appropriate when it is known from the outset she did not actively harm her child.^30^ If it is determined that a parent has been so wilfully negligent as to warrant punishment for being complicit in allowing abuse to continue, charging them with child cruelty^31^ is more appropriate than charging them with a homicide offence.

The current position of charging women with this homicide offence to force them to testify against their partner downplays the impact of being charged with section 5, on both the woman herself and her surviving children (Dunlap 2004). The average custodial sentence for women convicted of this offence is 44.6 months (3.7 years). Even if acquitted, the logic of using this offence coercively fails to acknowledge the impact of being charged with a homicide offence. As cases like *R v Green and Critchley* and *R v Rigby and Smedley* show, even where it is established from the outset which partner caused the harm to the child, the partner who “allowed” the harm will usually be held in custody until the trial. This has an enormous impact; a woman who has lost one child is then separated from any surviving children and wider family while she awaits trial. Critchley had already served approximately six months in prison before being tried. Time on remand is increasing given the current backlog of cases exacerbated by the ongoing COVID-19 pandemic. Mothers cannot maintain contact with surviving children at this time due to the restrictions regarding prison visitors. In England and Wales, all prison visits ceased entirely between the 24th March and 14th July 2020, and many still face restrictions; for example HMP Manchester allowed visits to resume in August but not for children under the age of 10 (Proctor 2020). There are also further reaching consequences of being charged with this offence. Even if women are ultimately acquitted, it has implications for future employment prospects and regaining custody of any surviving children. Due to the difference in evidential standard between the criminal and family courts, it is likely that a mother acquitted of failing to protect her child will not regain custody of her surviving children.

## Conclusion

This article has highlighted and analysed the striking reversal of usual patterns of prosecution and conviction around the offence of ‘causing or allowing a child (or vulnerable adult) to die or suffer serious physical harm’. In so doing, it has revealed the ways in which this offence criminalises women, particularly those who have suffered domestic abuse. An analysis of failure to protect cases in both the law reports and popular media underlines how prosecutors rely on narratives of victim-blaming and idealised motherhood to convince juries that the defendant foresaw or ought to have foreseen a risk of harm and failed to take reasonable steps to prevent it. My observations of *R v Green and Critchley* show how this downplays the striking situational vulnerability of defendants like Natalie Critchley. Critchley was not only vulnerable on account of the interpersonal violence and abuse she had suffered, her vulnerability was further exacerbated by her role as caregiver, (both in terms of responding to her children’s dependency and the responsibility which is associated with the status ‘mother’), and broader socio-economic precarity. This denial of the situational vulnerability of defendants who have experienced violence is paradoxical given that the Domestic Violence, Crime and Victims Act 2004 was generally intended to give new means of protection and redress to such women. Criminalising abused women for ‘allowing’ their children to come to harm obscures the reality that intimate partner violence remains endemic, not a rare social problem experienced by a small minority of women.

As I have demonstrated, when women are charged under section 5, prosecutors commonly seize upon a history of domestic abuse in order to prove that the defendant knew harm was foreseeable. My analysis of unreported case law confirms that the foreseeability of harm can more readily be established in the case of mothers due to conventional expectations of them that do not apply to fathers. This crime of omission is all too easily proven in a culture where mothers are expected to be omnipresent and are assumed to be responsible for every aspect of childcare and child safety. Moreover, I have shown how prosecutors rely upon archaic maternal ideals when trying to convince the jury that the defendant failed to take reasonable steps to prevent harm. The ‘reasonable steps’ expected of mothers demonstrate little understanding of the realities of domestic abuse; the prosecutorial inference that the only way an abused woman may protect her children is by leaving the relationship fails to acknowledge that the dissolution of the relationship is the most dangerous time for women and children (Jaffe et al. 2014). The precarious position of abused women charged with this offence is further exacerbated by section 6. This provision allows the CPS (and ultimately the jury) to conflate a failure to incriminate her partner with a mother’s failure to adequately protect their child, thereby silencing and denying her agency. To treat abused women as by-products of the criminal justice process in order to secure a murder conviction is ethically unjustifiable.

More generally this article demonstrates that, notwithstanding decades of feminist analysis, critique and protest, there is still much to be done, not only to eradicate gendered assumptions in reforming legislation, but also the way in which these assumptions are later seized on and manipulated in court to convince juries to convict. I have shown how misogynistic myths continue to influence the drafting of criminal offences resulting in a disjunct in the legislative process whereby an Act which ostensibly seeks to assist victims of abuse criminalises abused mothers. The cases analysed throughout this article then reveal that these gendered cultural assumptions are, in turn, consciously invoked by prosecutors when women are on trial as they continue to play to juries innate prejudices thus rendering abused mothers as the vulnerable victims of failure to protect legislation.

## Footnotes

1 The CPS stopped publishing their ‘outcomes by offence’ data tool (including breakdown of defendant by gender) at the end of 2018.

2 While men are also victims of intimate partner abuse, the rate and seriousness of violence and abuse is substantially lower than that suffered by women (Johnson 2010).

3 Reported cases are very scarce: as of October 2020 there have only been 11 reported cases involving a section 5 offence against a child, virtually all of which are appeals against sentencing.

4 *R v Green & Critchley* (Preston Crown Court (Sessions House), 10 April–24 May 2013).

5 A young child had died, and her father (Green) was charged with one count of murder and a second count of a section 5 offence and her mother (Critchely) was charged only under section 5.

6 (1986) 82 Cr App R 5.

7 Domestic Violence Crime and Victims (Amendment) Act 2012 s 1.

8 Domestic Violence, Crime and Victims Act 2004, s 5 as amended by the Domestic Violence, Crime and Victims (Amendment) Act 2012, s 1.

9 Or vulnerable adult.

10 Aside from the aforementioned peak in charges between 2015 and 2016.

11 UK Hansard 2004c, Domestic Violence Crime and Victims Bill Debate 24 June 2004, col. 95, per Dominic Grieve MP.

12 *R v Horncastle* [2010] 2 AC 373 at [83].

13 *R v Khan & others* [2009] EWCA Crim 2.

14 *R v Rigby & Smedley* (Manchester Crown Court, October 2012).

15 *R v Green & Critchley* (Preston Crown Court (Sessions House), 10 April–24 May 2013) Ruling of no case to answer para 18 v-vi (Author’s emphasis).

16 *LBC Sutton v Gray v Butler* [2012] EWHC 2604 (Fam).

17 *R v Green & Critchley* (Preston Crown Court (Sessions House) 23 March 2013).

18 *R v Khan & others* [2009] EWCA Crim 2.

19 Lord Judge CJ in *R v Khan & others* [2009] EWCA Crim 2 at [33].

20 *R v Thomas (Ashlea & Paul James)* (Birmingham Crown Court, April 2015).

21 *R v Green & Critchley* (Preston Crown Court (Sessions House), 10 April–24 May 2013).

22 *R v Stephens & another* [2007] EWCA Crim 1249.

23 *R v Ege (Sara)* [2014] EWCA Crim 772 (Cardiff Crown Court, January 2013).

24 *R v Green & Critchley* (Preston Crown Court (Sessions House), 10 April–24 May 2013).

25 *R v Lewis (Rebecca)* (Swansea Crown Court, May 2006).

26 *R v Kenny (Hayley*) (Chelmsford Crown Court, January 2007).

27 *R v Stephens & another* [2007] EWCA Crim 1249.

28 See also *R (Rights of Women) v Lord Chancellor and Secretary of State for Justice* [2016] EWCA Civ 91.

29 *R v Lane & Lane* (1986) 82 Cr App R 5.

30 Issues surrounding CPS charging decisions regarding this offence and other relatively new offences such as FGM suggests the CPS need clear guidelines on charging specific offences, particularly relative new offences which have gendered implications (See Berer 2020). Current CPS guidance mentions domestic abuse in a very tokenistic, vague sense in that it is something that prosecutors should be mindful about when assessing if a defendant took reasonable steps to mitigate against harm. It is not considered relevant to foreseeability (Home Office 2005a; Ministry of Justice 2012; CPS 2019b).

31 Contrary to Children and Young Persons Act 1933, s1.

## References

[CR2] Badinter, Elizabeth. 1980. *The myth of motherhood: an historical view of the maternal instinct*. London: Souvenir Press.

[CR3] Barlow Charlotte (2015). Silencing the other: Gendered representations of co-accused women offenders. The Howard Journal of Criminal Justice.

[CR4] Barnett Adrienne (2015). ‘Like gold dust these days’: Domestic violence fact–finding hearings in child contact cases. Feminist Legal Studies.

[CR5] BBC News Online. 2012. Yaseen Ali Ege murder trial: Sara Ege ‘beat son to death’. https://www.bbc.co.uk/news/uk-wales-south-east-wales-17916367. Accessed 4th April 2020.

[CR6] Bell Christine, Fox Marie (1996). Telling Stories of Women who Kill. Social and Legal Studies.

[CR7] Berer Marge (2020). Prosecution of female genital mutilation in the UK: Injustice at the Intersection of Good Public Health Intentions and the Criminal Law. Medical Law International.

[CR8] Bishop Charlotte, Bettinson Vanessa (2017). Evidencing domestic violence, including behaviour that falls under the new offence of ‘controlling or coercive behaviour’. The International Journal of Evidence and Proof.

[CR9] Brown Kate, Ecclestone Kathryn, Emmel Nick (2017). The many faces of vulnerability. Social Policy and Society.

[CR10] Cain Ruth (2016). Responsiblising recovery: lone and low paid parents, universal credit and the gendered contradictions of UK welfare reform. British Politics.

[CR11] Cain Ruth, Garrett Roberta, Jensen Tracey, Voela Angie (2016). ‘‘Just what sort of mother are you?’’ Neoliberal guilt and privatised maternal responsibility in twenty first century domestic crime fiction. We Need to Talk about Family: Essays on the Family, (Popular) Culture and Neoliberalism.

[CR94] Carlen Pat, Worrall Anne (1987). Gender Crime and Justice.

[CR12] Carlton Rosemary, Krane Julia, Strega Susan (2013). Take a Chance on Me. Failure to Protect: Moving Beyond Gendered Responses.

[CR13] Carter, Claire. 2015. Father jailed for 10 years for killing his ‘smiley, cheerful’ 11-month-old son who suffered injuries as bad as if he'd been in a 40mph car crash. *Mail Online,* 1 April.

[CR14] Chan Wendy, Rigakos George (2002). Risk, crime & gender. British Journal of Criminology.

[CR15] Choudhry Shazia (2019). When women's rights are not human rights – the non-performativity of the human rights of victims of domestic abuse within english family law. Modern Law Review.

[CR16] Clayton-Helm Lauren (2014). To punish or not to punish? Dealing with death or serious injury of a child or vulnerable adult. Journal of Criminal Law.

[CR17] Cobb Neil (2008). Compulsory caregiving: some thoughts on relational feminism, the ethics of care and omissions of liability. The Cambrian Law Review.

[CR18] Conaghan Joanne (2000). Reassessing the feminist theoretical project in law. Journal of Law and Society.

[CR19] Crown Prosecution Service 2018. Outcomes by Offences data tool.

[CR20] Crown Prosecution Service. 2019a. Partial Defences and Domestic Abuse Related Homicide in Homicide: Murder and Manslaughter – Legal Guidance, Violent Crime https://www.cps.gov.uk/legal-guidance/homicide-murder-and-manslaughter. Accessed 31st December 2018.

[CR220] Crown Prosecution Service. 2019b. Familial Deaths and Serious Physical Harm: Reasonable Steps in Homicide: Murder and Manslaughter – Legal Guidance, Violent Crime. http://www.cps.gov.uk/legal/h_to_k/homicide_murder_and_manslaughter/#familial. Accessed 1st January 2019.

[CR22] Cunliffe Emma (2011). Murder, Medicine and Motherhood.

[CR23] de Benedictis Sara (2012). Feral Parents: austerity parenting under neoliberalism. Studies in the Maternal.

[CR24] Dixon, Hayley. 2012. Mother jailed for letting her son die at the hands of her boyfriend who hit him so hard his spleen split in two. *Daily Mail,* 21 December.

[CR25] Dobson, Charlotte. 2014. Chilling window into violent relationship of murdered toddler’s parents. *The Bolton News*, March 20, 2014.

[CR26] Dunlap Justine (2004). Sometimes I feel like a motherless child: The error of pursuing battered mothers for failure to protect. Loyola Law Review.

[CR27] Edwards Susan (2012). The duplicity of protection prosecuting frightened victim: An act of gender based violence. Journal of Criminal Law.

[CR28] Elkin, Meghan. 2019. The nature of violent crime in England and Wales: Year ending March 2018. https://www.ons.gov.uk/peoplepopulationandcommunity/crimeandjustice/articles/thenatureofviolentcrimeinenglandandwales/yearendingmarch2018. Accessed 4th April 2020.

[CR29] Enos V (1996). Prosecuting attered mothers: State laws’ failure to protect women and children. Harvard Women’s Law Journal.

[CR30] Freer Elaine (2016). Causing or Allowing the Death of a Child: challenges to working out “which of you did it?”. Criminal Law Review.

[CR31] Friedman Marilyn, Mackenzie Catriona, Rogers Wendy, Dodds Susan (2014). Moral Responsibility for Coerced Wrongdoing: The Case of Abused Women Who “Fail to Protect” Their Children. Vulnerability: New Essays in Ethics and Feminist Philosophy.

[CR100] Fugate Jeanne (2001). Who’s failing whom? A critical look at failure to protect laws. New York University Law Review.

[CR32] Gardner Carol (1995). Passing by Gender and Public Harassment.

[CR33] Goc, Nicola. 2007 Monstrous Mothers and the Media. In *Monsters and the Monstrous: Myths and Metaphors of Enduring Evil.* ed. Niall Scott. New York: Rodopi.

[CR34] Hannah-Moffat Kelly (2009). Gridlock or mutability: reconsidering “gender” and risk assessment. Criminology & Public Policy.

[CR35] Harrison Christine (2008). Implacably hostile or appropriately protective? women managing child contact in the context of domestic violence. Violence Against Women.

[CR36] Hayes Mary (2005). Criminal trials where a child is the victim: Extra protection for children or a missed opportunity?. Child & Family Law Quarrterly.

[CR37] Herring Jonathan, Cunningham Sally, Clarkson Christopher M V (2008). Mum’s not the word: An analysis of section 5, Domestic Violence, Crime and Victims Act (2004). Criminal Liability for Non-Aggressive Death.

[CR38] Herring, Jonathan. 2007. Familial homicide, failure to protect and domestic violence: Who’s the victim? *Criminal Law Review* December: 923–933.

[CR39] Home Office. 2005a. The Domestic Violence Crime and Victims Act 2004. https://www.gov.uk/government/publications/the-domestic-violence-crime-and-victims-act-2004. Accessed 30 December 2018.

[CR221] Home Office. 2005b. The Domestic Violence, Crime and Victims Act. https://www.gov.uk/government/publications/the-domestic-violence-crime-and-victimsact-2004. Accessed 4th May 2020.

[CR41] Jacobs Michelle (1998). Requiring Battered Women Die: Murder Liability For Mothers Under Failure To Protect Statutes. The Journal of Criminal Law and Criminology.

[CR42] Jaffe Peter, Campbell Marcie, Olszowy Laura, Hamilton Leslie (2014). Paternal filicide in the context of domestic violence: Challenges in risk assessment and risk management for community & justice professionals. Child Abuse Review.

[CR43] Johnson Michael (2010). A Typology of Domestic Violence: Intimate Terrorism, Violent Resistance, and Situational Couple Violence.

[CR44] Karamessini Maria, Rubery Jill (2014). Women and Austerity: The Economic Crisis and the Future for Gender Equality.

[CR45] Keen, Liz. 2013. Yousef Ali Ege: ‘My son Yaseen never complained to me’. *Wales Online* 26th March. https://www.walesonline.co.uk/news/wales-news/yousuf-ali-ege-my-son-2029032. Accessed 4 April 2020.

[CR46] Kyd Sally, Elliott Tracy, Walters Mark (2017). Clarkson and Keating: Criminal Law.

[CR200] Law Commission. 2003. *Children: Their Non-Accidental Death or Serious Injury* (Criminal Trials) (Law Com Report No. 282).

[CR47] Lancashire Post. 2013. Lia’s mum told lover her partner was innocent. *Lancashire Post,* 20 April.

[CR48] Lapierre Simon (2008). Mothering in the context of domestic violence: The pervasiveness of a deficit model of mothering. Child and Family Social Work.

[CR49] Leveillee Suzanne, Marleau Jacques, Dube Myriam (2007). Filicide: A Comparison by Sex and Presence or Absence of Self-Destructive Behaviour. Journal of Family Violence.

[CR50] Magen Randy (1999). In the Best Interests of Battered Women: Reconceptualising Allegations of Failure to Protect. Child Maltreatment.

[CR51] Mahoney Martha (1991). Legal images of battered women: Redefining the issue of separation. Michigan Law Review.

[CR53] Mezey Gillian, Bewley Susan (1997). Domestic violence and pregnancy: Risk is greatest after delivery. British Medical Journal.

[CR54] Miccio G. Kristian (1999). A Reasonable Battered Mother? Redefining, Reconstructing and Recreating the Battered Mother in Child Protective Proceedings. Harvard Women’s Law Journal.

[CR55] Midson Brenda (2014). The Helpless Protecting the Vulnerable? Defending Coerced Mothers Charged with Failure to Protect. Victoria University of Wellington Law Review.

[CR56] Ministry of Justice. 2011. Publicising Sentencing Outcomes. https://assets.publishing.service.gov.uk/government/uploads/system/uploads/attachment_data/file/487464/20150413-Publishing_Sentencing_Outcomes_MoJ_Guidance_HQMCSPA-O.pdf. Accessed 6 February 2021.

[CR57] Ministry of Justice. 2012. Domestic Violence, Crime and Victims (Amendment) Act 2012, Circular No. 2012/03 29 June.

[CR101] Moffat, Andy. 2018. Ellie May death Trial: Sick couple blamed each other for toddler’s death. *Lancashire Post*, 16th November.

[CR58] Morrison Samantha (2013). Should There be a Domestic Violence Defence to the Offence of Familial Homicide?. Criminal Law Review.

[CR60] Nadler Janice (2012). Blaming as a Social Process: The Influence of Character and Moral Emotion on Blame. Law and Contemporary Problems.

[CR61] Naffine Ngaire (1997). Feminism and Criminology.

[CR62] Nicolson Donald (1995). Telling tales: Gender discrimination, gender construction and battered women who kill. Feminist Legal Studies.

[CR64] Office for National Statistics. 2019. Families and Households. https://www.ons.gov.uk/peoplepopulationandcommunity/birthsdeathsandmarriages/families/bulletins/familiesandhouseholds/2019. Accessed 4 April 2020.

[CR65] Panko Linda (1995). Legal backlash: The expanding liability of women who fail to protect their children from their male counter parts abuse. Hastings Women’s Law Journal.

[CR67] Porter Antonia (2019). Prosecuting domestic abuse in england and wales: crown prosecution service ‘working practice’ and new public managerialism. Social & Legal Studies.

[CR68] Proctor, Kate. 2020. Ban of prison visits in England and Wales breaches children’s rights, say lawyers. *The Guardian,* 31 July.

[CR69] Quick Oliver, Wells Celia (2006). Getting tough with defences. Criminal Law Review.

[CR70] Rabin, Bonnie. 1995. Violence Against Mothers Equals Violence Against Children: Understanding the connections. *Albany Law Review* 58(4): 1109–1117.

[CR102] Reed Alan (2010). Spousal Homicide and Foresight of Harm. Journal of Criminal Law.

[CR71] Reiner Robert (2007). Success or statistics? New Labour and Crime Control: What happened to crime under new labour?. Criminal Justice Matters.

[CR72] Seal Lizzie (2010). Women, Murder and Femininity: Gender Representations of Women Who Kill.

[CR1] Singh Sarah (2017). Criminalising vulnerability: protecting ‘vulnerable’ children and punishing ‘wicked’ mothers. Social and Legal Studies.

[CR73] Skinazi Heather (1997). Not Just a Conjured Afterthought; Using Duress as a Defence for Battered Women who Fail to Protect. California Law Review.

[CR74] Smart Carol, Silva E (1996). Deconstructing Motherhood. Good Enough Mothering? Feminist Perspectives on Lone Motherhood.

[CR75] Smith, Tom, and Ed Cape. 2017. The rise and decline of criminal legal aid in England and Wales. In *Access to Justice & legal Aid: Comparative Perspectives on Unmet Legal Aid,* ed. Asher Flynn and Jacqueline Hodgson. 63–86. Oxford: Hart.

[CR76] Stanko Elizabeth A (1997). Safety talk: Conceptualising Women’s Risk assessment as a ‘Technology of the Soul’. Theoretical Criminology.

[CR77] Stark Evan (1995). Re-presenting woman battering: From battered woman syndrome to coercive control. Albany Law Review.

[CR78] Stubbs Julie, Fitz-Gibbon Kate, Walklate Sandra (2016). Murder, Manslaughter and Domestic Violence. Homicide, Gender and Responsibility.

[CR79] United Kingdom Hansard. 2004a. House of Commons Debates. Vol. 422. 14 June. Col 69–77.

[CR80] United Kingdom Hansard. 2004b. House of Lord Grand Committee. Vol. 657. 21 January.

[CR81] United Kingdom Hansard. 2004c. House of Commons Standing Committee E. 22 June.

[CR82] United Kingdom Hansard. 2004d. House of Lords Debates. Vol. 658. 9 March.

[CR83] Walklate Sandra (1997). Risk and Criminal Victimisation: A Modernist Dilemma. British Journal of Criminology.

[CR84] Wallbank Julie (1998). Castigating mothers: The judicial response to “wilful” women in disputes over paternal contact in English law. Journal of Social Welfare and Family Law.

[CR85] Ward Tony (2004). Experts, Juries and Witch Hunts: From Fitzjames Stephen to Angela Cannings. Journal of Law and Society.

[CR86] Weait Matthew, Munro Vanessa E, Stychin Carl (2007). On Being Responsible. Sexuality and the Law: Feminist Engagements.

[CR87] Weare Siobhan (2013). ‘The mad’, ‘the bad’, ‘the victim’: Gendered constructions of women who kill within the criminal justice system. Laws.

[CR88] Weisz Arlene, Wiersma Rebecca (2011). Does the public hold abused women responsible for protecting children? Affilia: Journal of Women and Social. Work.

[CR89] Women’s Aid. 2016. Nineteen Child Homicides. https://1q7dqy2unor827bqjls0c4rn-wpengine.netdna-ssl.com/wp-content/uploads/2016/01/Child-First-Nineteen-Child-Homicides-Report.pdf. Accessed 4 April 2020.

[CR90] Women’s Budget Group. 2019a. Triple Whammy: The impact of local government cuts on women. https://wbg.org.uk/wp-content/uploads/2019/03/Triple-Whammy-the-impact-of-local-government-cuts-on-women-March-19.pdf. Accessed 4 April 2020.

[CR91] Women’s Budget Group. 2019b. Benefits or Barriers? Making Social Security Work for Survivors of Violence and Abuse Across the UK’s Four Nations. https://wbg.org.uk/wp-content/uploads/2019/06/Benefits-or-barriers-4-nations-report.pdf.. Accessed 4 April 2020.

[CR93] Worden Alissa P, Carlson Bonnie E (2005). Attitudes and beliefs about domestic violence: results of a public opinion survey: beliefs about causes. Journal of Interpersonal Violence.

[CR95] Worrall Anne (1990). Offending Women: Female Lawbreakers and the Criminal Justice System.

